# Defining and Diagnosing Obesity in India: A Call for Advocacy and Action

**DOI:** 10.1155/2023/4178121

**Published:** 2023-11-07

**Authors:** Sanjay Kalra, Nitin Kapoor, Madhur Verma, Shehla Shaikh, Sambit Das, Jubbin Jacob, Rakesh Sahay

**Affiliations:** ^1^Department of Endocrinology, Bharti Hospital, Karnal, Haryana, India; ^2^Department of Endocrinology, Diabetes and Metabolism Christian Medical College & Hospital, Vellore, Tamil Nadu, India; ^3^Department of Community/Family Medicine, All India Institute of Medical Sciences, Bhatinda, Punjab, India; ^4^Prince Aly Khan Hospital, Mumbai, Maharashtra, India; ^5^Department of Endocrinology, Kalinga Institute of Medical Sciences (KIMS), Bhubaneswar, Odisha, India; ^6^Department of Endocrinology, Christian Medical College, Ludhiana, Punjab, India; ^7^Department of Endocrinology, Osmania Medical College, Hyderabad, Telangana, India

## Abstract

The prevalence of overweight and obesity has more than doubled since 1980, and it is predicted that around two-thirds of the global burden of the disease will be attributed to chronic non-communicable diseases. Developing countries are experiencing a more dramatic rise in the prevalence of obesity in recent years. As per National Family Health Survey-5 (NFHS-5), one in every four Indians is now having obesity. It has been reported that being overweight and obese is a significant problem among different socioeconomic spectrums of men and women in India, especially among the elderly, people residing in urban regions, and diverse socioeconomic strata. There is an urgent need to identify obesity as a chronic disease requiring immediate attention, mandating timely screening, timely treatment, and economical ways of achieving and managing weight loss across the country. In this review, the authors have discussed various aspects of overweight and obesity and critically appraised the current status of obesity in India, its public health implications, the significance of screening, the role of BMI and other parameters in diagnosing obesity, and the need for treatment and cost-effective prescriptions.

## 1. Introduction

As per the World Health Organization (WHO) estimates, 1.9 billion adults worldwide were overweight in 2016, while 650 million individuals had obesity [1]. Weight gain resulting in overweight and obesity is one of the most important factors behind non-communicable diseases. Around the globe, health systems are experiencing the economic burden of the rising prevalence of overweight and obesity. Successful weight loss interventions for high-risk populations will help reduce health and economic costs [2]. There is a need to identify obesity as a chronic disease that requires immediate attention, proper screening, treatment, and cost-effective ways to sustain weight loss. In this white paper, endocrinology experts have reviewed the current status of obesity in India and discuss in detail the epidemiology of obesity, its public health significance, the definition of obesity, whether the definition of obesity fits the purview of disease, screening, and assessment, its clinical implications, and the need for treatment and cost-effective prescriptions.

## 2. Defining Obesity

*Does It Fit the Classification of Disease vs. Epidemic?* Obesity or overweight is a chronic disease resulting from an interaction between an individual's genetic predisposition to weight gain and environmental influences. Overweight and obesity occur when excess fat accumulation increases the risk to health [3]. WHO defines overweight and obesity as “abnormal or excessive fat accumulation that presents a risk to health.” It is a chronic, relapsing progressive disease defined by excessive adiposity that may impair health [4]. The WHO defines overweight as a BMI greater than or equal to 25 kg/m^2^, while obesity is a BMI greater than or equal to 30 kg/m^2^ [5]. In a 2000 consensus, a WHO expert group proposed the BMI criterion for overweight as 23–24.9 kg/m^2^ and obesity as ≥25 kg/m^2^ for individuals in the Asia-Pacific region [6]. In 2009, more than 100 Indian medical experts brain-stormed and published consensus guidelines defining overweight as those with a BMI between 23.0 and 24.9 kg/m^2^ and obesity as those having a BMI ≥25 kg/m^2^ [7]. As per the WHO's data, the global prevalence of obesity almost tripled between 1975 and 2016, with a considerable increase in most of the world's nations, including those in the low-income and middle-income categories. The global spread of obesity has been labeled a pandemic [8].  Table 1 shows the definition of obesity per various organizations worldwide [4, 9–19]. Considering all these aspects, we define obesity as “a chronic multifactorial and multidimensional syndrome characterized by an inappropriate percentage or distribution of adipose tissue associated with multisystemic comorbidities and complications, which require long-term mitigation and management.” Algorithm 1 provides some important facts about health and obesity.

## 3. Obesity Epidemiology

Globally, the prevalence of obesity has tripled since 1975 and has been universally labeled as a pandemic by experts [9]. As per a WHO report, at least 1 in 3 of the world's adult population is overweight, and almost 1 in 10 is obese [21]. With some lower-income countries showing the highest increases in the last decade, no country has reported a decline in obesity prevalence across their entire population, and none are on track to meet the World Health Organization's target (WHO's target) of no increase in 2010 levels by 2025. The estimates for global levels of overweight and obesity (BMI ≥ 25 kg/m^2^) suggest that over 4 billion people may be affected by 2035, compared with over 2.6 billion in 2020. This reflects an increase from 38% of the world's population in 2020 to over 50% by 2035. The prevalence of obesity (BMI ≥ 30 kg/m^2^) alone is anticipated to rise from 14% to 24% of the population over the same period, affecting nearly 2 billion adults, children, and adolescents by 2035 [22].

Like every other country affected by overweight and obesity, India is also witnessing a drastic increase in the prevalence. As per World Obesity reports, the prevalence of obesity (BMI ≥ 24 kg/m^2^) is around 11%, which is much higher than that in other low-middle-income countries and is closer to the estimated in high-middle-income countries. The most recent round of the National Family Health Survey-5 (2019–2021) covered 636 699 households, with 724115 women (aged 15–49) and 101839 men (aged 15–54). NFHS is an Indian version of the Demographic Health Survey that collects, analyses, and disseminates representative data on population health and nutrition in over 90 countries using nationally representative surveys. As per the analysis of the survey (based on WHO Asia-Pacific guidelines), the overall weighted prevalence of overweight and obesity (BMI ≥ 22.9 kg/m^2^) in the male and female study participants as per NFHS-5 was 44.02% and 41.16%, compared to 37.71% and 36.14% in NFHS-4, depicting a percentage relative increase of 16.7% and 13.8% [23]. The prevalence also depicts significant sociodemographic variations similar to our observations in NFHS-4, with wide geographical disparities. It was seen that all states, northern, western, southern, and most of the northeastern states of India, demonstrated a higher prevalence of overweight and obesity than the national average for males and females. On the other hand, the Central and East Indian states showed a lower prevalence of obesity or overweight [24]. The data also suggest a typical urban-rural divide [24, 25]. Similarly, the results from the first wave of the Longitudinal Ageing Study in India (2017-18), which is another nationally representative survey of over 73 000 older adults aged 45 and above across all states and union territories of India, depicted that the overall prevalence of overweight and obesity (BMI > 22.9 kg/m^2^) was 14.02% (*n* = 8, 283) and 27.1% (*n* = 16, 022), respectively. Contrary to NFHS findings, the results from LASI depict that a significant proportion of older females are obese (32.0%) compared to older males (21.4%) [26]. The estimates from these national surveys are coherent with the ICMR-INDIAB study, conducted among adults aged 20 years and older,which estimated the overall weighted prevalence of generalized obesity (BMI of 25 kg/m^2^) and abdominal obesity (waist circumference of 90 cm or higher for men and 80 cm or higher for women) to be 28.6% (26.9–30.3; 29861 of 110 368 individuals) and 39.5% (37.7–41.4; 40 121 of 108 665 individuals), respectively [27].

While most estimates are for adults, national-level estimates are scarce for children and adolescents when we know that the rising prevalence of global obesity is expected to be steepest among children and adolescents, rising from 10% to 20% among boys and from 8% to 18% among girls during the period between 2020 and 2035 [22]. To fill in the gap, Comprehensive National Nutrition Survey (CNNS)-2018-19 was conducted in India to provide the most comprehensive picture of the nutritional status of pre-schoolers (0–4 years), school-age children (5–9 years), and teenagers (10–19 years) with a total sample size of 112, 245. In a secondary data analysis that included 65562 participants in which biochemical parameters were collected, the prevalence of overweight and obesity was estimated to be 2.69%, 4.18%, and 4.99%, respectively [28] Cardiometabolic risk factors are observed in normal-weight Asian Indian children, and the abnormalities are higher in overweight children [25].

### 3.1. Variations in Obesity

In addition, a significant proportion of the South Asian phenotype may have high body fat despite a normal body mass index (BMI), a phenotype called normal weight obesity [29, 30]. In a study from the Indian state of Kerala, about a third of the population had normal weight obesity [31]. Then, another frequent variation of obesity is referred to as sarcopenic obesity, which is defined as the co-existence of obesity and sarcopenia. The diagnosis of sarcopenic obesity depends on the accompanying increased BMI or waist circumference with ethnicity-specificity cut points along with surrogate indicators of sarcopenia. Sarcopenia is an age-associated, involuntary loss of skeletal muscle mass and strength. It may even begin in the fourth decade of life; it has been suggested that skeletal muscle mass and strength reduce linearly, with about 50% of reductions of muscle mass being lost by the individual reaches the age of 80 [32].

### 3.2. Surrogate Indicators of Obesity

**Figure 1:**
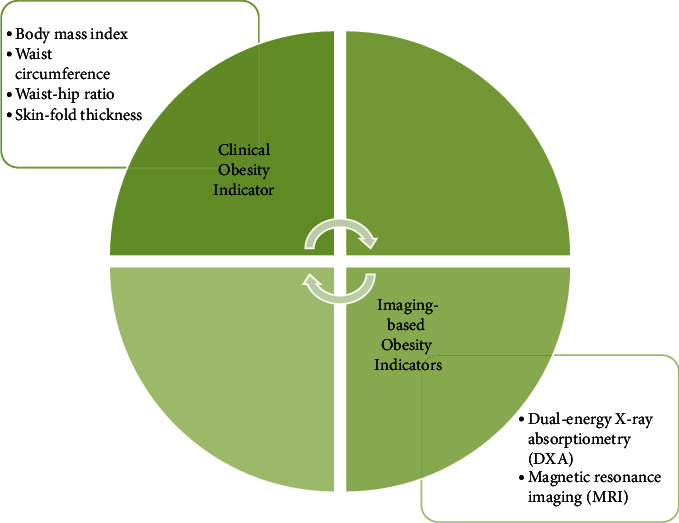
Clinical and imaging indicators of obesity.

In addition to the conventional indicators like BMI, waist circumference, waist-hip ratio, neck circumference, waist-height ratio, and body fat estimation are also added to the diagnostic markers.  Table 2 depicts the different markers and their values used for evaluating obesity. More sophisticated imaging can also be used for visceral fat estimation, which is considered to be the most reliable obesity indicator to assess the underlying cardiometabolic risk factors accurately [33].  Figure 1 depicts the clinical and imaging-based obesity indicators.

### 3.3. Socioeconomic Disparities in Obesity Prevalence

A recent study has shown a significant increase in the rate of overweight/obesity among adults in India in a short time, and it is attributable to changes in the sociodemographic characteristics, including a rapid transition in their lifestyle, work culture, substance abuse, reduced physical activity, and unhealthy eating practices. The major contributors to overweight and obesity are age, urban residence, wealth index, access to clean fuel, toilet facilities, and unhealthy diet [34]. BMI in male and female populations is also affected by two independent factors: ethnicity and socioeconomic status. Individuals with partial ancestry in high-risk groups have an intermediate risk for obesity. Certain ethnic groups are especially susceptible to the adverse health effects of obesity. A study has shown that Indian males were at higher risk of being overweight than white British males. An Indian study assessing the link of ethnicity with overweight and obesity from different geographical regions of India showed that general body fat deposition was found to be highest among Delhi females and males [35]. Luke et al. showed that environmental factors are the major drivers for ethnic differences in obesity [36]. Kapoor et al. further stressed and observed that ethnic differences were obvious among the South Indians, North Indians, and Northeast Indians pertaining to obesity and its association with cardio-respiratory health [35]. The ethnic differences in adiposity and the intermediate values in admixed individuals may stem from genetic and environmental sources [37]. Apart from the genetic composition, the variation in body composition is due to differences in energy intake and physical activity. Body composition, including fat vs. muscle and abdominal visceral fat vs. abdominal subcutaneous fat, may differ between ethnic groups due to genetic differences [38]. The INDIA-ICMR study in 2014 depcited high physical inactivity in India (54.5%), and inactivity was higher in urban areas (60%)compared to rural (50%), and also more among women than men [39].

## 4. Obesity as a Public Health Issue

Overweight and obesity cause over 3.4 million deaths, 4% of years of life lost, and at least 4% of disability-adjusted life years worldwide [40]. The epidemiology detailed above clearly shows that the high burden of overweight and obesity in India results in a significant impact across diverse settings. The issue is costing the Indian economy an annual expenditure of $23.2 billion, which is a total cost of 1.7% of the gross domestic product (GDP) in India, which is based on a sensitivity analysis valuing life expectancy gains from avoiding premature mortality at a multiple of GDP. Without proper public health measures such as screening, treatment, and awareness programs, the country may suffer a loss of almost $440 billion, a 19-fold increase in obesity costs by 2060. India ranks third in the projected economic burden attributed to overweight and obesity, next to China and the USA [41]. A report by the World Obesity Federation in 2017 said that India will spend US$13 million annually for treating obesity-related illnesses by 2025 [42]. Apart from the direct medical costs incurred by the country, the issue of overweight and obesity is also linked with indirect costs associated with the process of seeking medical healthcare, economic loss from premature mortality, absence from workdays, and negative influence on work productivity. Direct costs comprise 32% of the total expenditure on obesity, while indirect costs contribute to 68% of the total obesity cost [43]. The estimated projection is that the prevalence of obesity will increase to about 57% of the population in India. As measured in 2019, the average GDP growth was 5% in India, while the estimated obesity cost ranged between 0.8% and 2.42% of GDP in 2019 in eight countries, including Australia, Brazil, India, Mexico, Saudi Arabia, South Africa, Spain, and Thailand. Clearly, the economic impact of obesity can be viewed as a significant hindrance to a country's economic development [41]. Yoong stated in his review that “actual societal cost savings are likely to be significantly higher than estimated” [2].

It has been reported in a study by Retat et al. that screening and referral behavioral weight management opportunistically endorsed, offered, and facilitated to patients results in reduced healthcare costs and health improvement [44]. The National Programme for Prevention and Control of Cancer, Diabetes, Cardiovascular Diseases and Stroke (NPCDCS), Ministry of Health and Family Welfare, has recommended screening, medical examination, and referrals for early interventions for high blood pressure or excess weight and obesity to reduce risk factors from becoming complicated and developing into non-communicable diseases [45]. Besides, obesity prevention and management efforts in a pediatric group require routine healthcare provider screening for BMI and social determinants of health to alleviate the causes leading to obesity. The guidelines recommend annual screening for excess weight using BMI in children as young as 2 to 6 years old. Dietary weight and physical activity counseling are recommended for all children and adolescents who are either overweight or obese [46].

## 5. Obesity Grading

The two common thresholds used to diagnose overweight and obesity are BMI cutoffs and increasing weight thresholds [3]. BMI is a statistical index utilizing a person's weight and height to assess body mass in males and females of any age. The international cutoffs are slightly changed for the Asian and South Asian populations, as they underestimate the obesity risk [6, 47].  Table 3 shows the obesity gradation based on BMI. The American Diabetes Association (ADA) position statement states that for screening for type 2 diabetes mellitus in Asians, a BMI cutoff of 23 kg/m^2^ is appropriate. Besides, the ADA has defined BMI cutoffs for screening for diabetes for Asian Americans as well as India and other Asian countries with lowered BMI cut points to define overweight and obesity. It has become increasingly clear that with the high prevalence of type 2 diabetes mellitus and cardiovascular risk factors in parts of Asia, including Hong Kong, Singapore, China, and India, it is important to consider the higher percentage of body fat and risk of cardiovascular disease at this BMI cutoff compared to Caucasians of the same demography [17, 47].  Table 3 depicts the classification of diabetes as per BMI in different regions worldwide.

However, despite an Indian guideline defining overweight and obesity since 2009, most researchers and clinicians in India refer to the international criteria for overweight and obesity, which may lead to serious health issues (refer Algorithm 2). When the patients are classified as non-obese, even when actually obese, they are at an increased risk of developing obesity-related diseases [48]. Hence, it is important to recognize obesity and overweight as a disease state that needs assessment and addressal.

## 6. Obesity Evaluation

**Figure 2:**
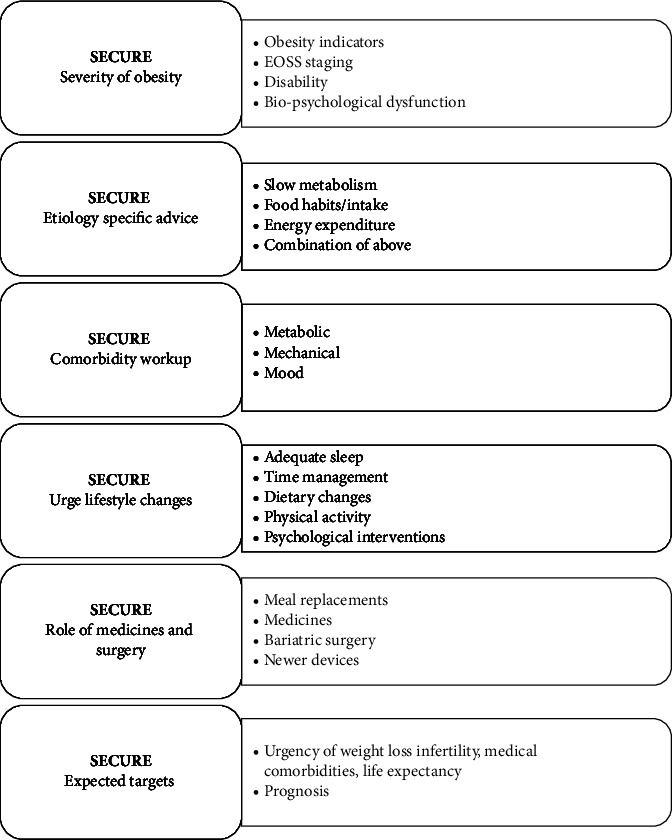
The SECURE model.

While BMI is primarily used as a means of classifying obesity alone, it is insufficient for the classification of a person as obese or malnourished [6, 49]. While BMI is a commonly used metric in adults for classifying obesity, it is a poor indicator of the percent of body fat. Besides, the BMI does not capture information on the mass of fat in different body sites [50]. Classification of obesity is based on measuring fat mass, percent body fat, and BMIs. The Edmonton Obesity Staging System (EOSS) is a five-stage system of obesity classification considering the metabolic, physical, and psychological parameters, which can guide the optimal obesity treatment. As per EOSS, patients in the more severe stages should be recommended for weight loss treatments; however, there are unclear benefits of treating obesity in those in the lower stages of EOSS [51]. The EOSS allows evaluating the effect of obesity-related comorbidities on individuals beyond weight [52]. In an Indian setting, data from a multidisciplinary obesity clinic revealed that about 68% of individuals belong to EOSS Stage 2 and 3 [53]. Kalra et al. have proposed a barophenotypic characterization to diagnose and describe obesity in a customized manner. It can be defined as “the sum of all attributes, both biophysical and social, which contributes to the overall impact of obesity on health.” The barophenotype is portrayed by an ABCDE rubric [54].  Table 4 depicts the barophenotypic characterization. Kapoor et al. proposed the SECURE model in 2020, which is a simplified yet systematic approach to classifying an obese patient with the aim of facilitating personalized treatment. SECURE model is “an alliterative six-item rubric” [55].  Figure 2 depicts the SECURE model.

## 7. Impact of Obesity

**Figure 3:**
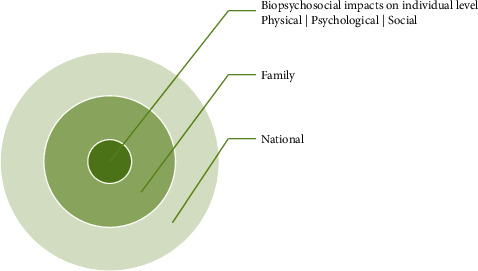
Impact of obesity on health.

Obesityhas deleterious impact on an indivudual, family, society, and the nation through a multifactorial pathway.  Figure 3 enumerates the impact of obesity on human health and life.

### 7.1. Biopsychosocial Impact

#### 7.1.1. Physical Health

The comorbidities associated with obesity and overweight include type 2 diabetes, hypertension, stroke, coronary artery disease, congestive heart failure, asthma, chronic back pain, osteoarthritis, cancers, pulmonary embolism, gallbladder disease, and a raised risk of disability. The comorbidities linked with obesity and overweight have been known to lead to 3 million deaths worldwide [40]. Obesity and overweight in childhood and adolescence are subjected to an increased risk of premature morbidity and mortality, especially cardiometabolic morbidity [56]. Disability due to obesity in type 2 diabetes might also increase due to arteriosclerosis, nephropathy, and retinopathy. In addition, an increasing prevalence of obesity will also significantly raise obesity-related morbidity and disability [57]. Obesity is a major public health issue resulting in reduced life expectancy, particularly in younger age groups. Other studies have also shown that overweight and obesity are significant problems for minorities, poor populations, and women [58]. It has been shown that Asian Indians are at a higher risk of cardiovascular risk factors and type 2 diabetes mellitus even at lower BMI levels than their white Caucasian counterparts [7, 59]. Indians are reported to have greater total truncal, intra-abdominal, subcutaneous, and ectopic tissue fat at a specific BMI level compared to Caucasians [7]. Studies have also revealed that obesity may increase the risk of poor sexual health. Some researchers have attributed these effects on sexual health to appearance and weight and encountering difficulties in sexual activities. The impaired sexual health often results in reduced sexual satisfaction, unintended pregnancy, and abortion. In the case of women requiring therapeutic procedures, their sexual health is significantly affected [40].

#### 7.1.2. Psychological Impact

A big challenge with obesity is that being overweight is a stigma in our society, and discrimination due to obesity can lead to mental issues. Some mental health issues triggered by obesity and overweight are low self-esteem, mood disorders, motivational disorders, eating problems, issues with body image, and negative impact on interpersonal communication. The mental health issues related to obesity result in a poor quality of life [58, 60]. Several studies from the Indian setting have reiterated the same [61, 62].

#### 7.1.3. Social Impact

Overweight and obesity are faced with a considerable health burden with a major impact on health expenditures. Due to the strong association of obesity with the incidence of chronic medical problems, there is an associated impairment of health-related quality of life, increasing healthcare and medication spending. The healthcare costs for obesity-related problems are substantially high for individuals as well as the healthcare systems of the country [40].

### 7.2. Impact on Family

The stigmatization associated with obesity and overweight affects all the crucial aspects of life, including growth and development, educational process, employment structure, and healthcare provision. Obese individuals not only get ridiculed but are also made to suffer from discrimination, social bias, rejection, and humiliation. Such discrimination is not only related to poor health behavior but also a determinant for psychological aberrations such as pathological overeating, binge eating, or even sedentary life and reduced physical activity, eventually resulting in more weight gain [63].

### 7.3. Impact on the Nation

Obesity is known to be responsible for 1% to 3% of total health expenditure in most countries, and costs are expected to rise alongside the rapid rise in obesity incidence and prevalence [64]. An Indian study conducted in Delhi has mentioned that obese women spend 2% to 3% more on health towards total health expenditure. Throughout their lives, healthcare expenditures for obese people are more than 25% compared to those with normal weight. Obesity has far-reaching implications for health systems and affects individuals and their families [65].

## 8. Need for Screening and Investigations

Asian individuals have lower BMI but high total and central adiposity, making them more vulnerable to metabolic diseases. Metabolic obesity is reported to be common in Asians [6]. Considering the health risks of overweight and obesity and its increasing prevalence, it is important to prevent obesity and the serious health-related outcomes and consequences. Obesity, overweight, and their impact on various aspects of health form one of the most important public health priorities [57]. Referring individuals to screen for obesity will lead to behavioral interventions to improve weight status and other risk factors for important health outcomes [66]. Screening in children and adolescents will enable clinicians to identify children requiring a thorough evaluation of obesity risk and to provide appropriate and timely counseling. In fact, it has been suggested that healthcare visits offer a well-timed and beneficial opportunity for clinicians to detect excess weight. This enables the clinician to offer patient counseling, inform them about associated health risks, and initiate management or treatments as the patient's condition demands [67].

## 9. Need for Treatment

While obesity is a severe global problem, it is also associated with a simultaneous increase in comorbidities. The weight loss results in reduced blood pressure, improved lipids, and reduced diabetes incidence. Hence, it becomes imperative to manage and treat obesity in its nascent stage [68]. It is important to know that the cost of obesity increases not only due to comorbidities but also due to absenteeism, disability, early death, and other factors attributed to obesity. Obesity counseling and treatment are beneficial for preventing and treating obesity. Since obesity is an issue of pandemic proportions and the cost of obesity and associated comorbidities are rising globally, more aggressive and effective prevention and treatment strategies are needed to address obesity [69]. Weight loss is recommended in overweight individuals, especially in the presence of other risk factors. Severely overweight or obese people, those with a BMI of 30 kg/m^2^ or more, are at an increased risk of disease irrespective of other risk factors, and weight loss is recommended for them.

## 10. Need for Cost-Effective Prescription

As obesity is an issue of public health concern, it is of considerable importance that cost-effective prevention and treatment of obesity are aggressively undertaken [69]. In treating obesity, interventions, including a low-fat diet with exercise, are of comparable cost to anti-obesity treatments such as orlistat [70]. However, there are not enough studies to assess the cost-effectiveness of the treatment program. It has been suggested that family or group treatments are better than individual treatment [69]. It has been reported that metabolic surgery is a more cost-effective alternative for severely obese individuals and those with obesity-related comorbidities [71]. On the other hand, the effect of weight loss was around 3 to 5 kg weight reduction with medical treatment spread over a duration of 6 months to 2 years [72]. However, further research is needed to make a more definitive assessment regarding the cost-effectiveness of a particular treatment.

## 11. The 4As of Obesity

1. Awareness: There is a substantial economic burden of obesity for individuals and their households, mandating an urgent intervention in obesity awareness and health promotion among Indian women. Prevention is definitely better than cure, and there is a need to raise awareness about the issue of obesity and develop community programs for its prevention among Indian women.
2. Attention: clinicians should screen for obesity in individuals 6 years and older and refer them or offer comprehensive, intensive behavioral interventions to promote improvements in weight status.
3. Advocacy: We know that classifying an individual as lean when the individual is truly obese may put them at risk for diseases associated with obesity and also delay a possible beneficial therapy. It has been shown that with accurate cutoff points and timely screening, an additional 10% to 15% of the population in India would be labeled as overweight or obese and require appropriate management. If country-wise guidelines are used for the gradation of obesity, it will reduce the rate at which the incidence of obesity, type 2 diabetes, and cardiovascular diseases is rising in the country, giving a definite boost to stabilizing health economics.
4. Addressal: As the discussion above clearly shows, the impact of obesity on the economics and health of the nation cannot be ignored, and it is important to address the root cause of the issue. The health fraternity, public health officials at the state and national levels, and policymakers should join hands together to consider the issue seriously. Public and community health measures should include population-based screening, obesity treatment, promotion of healthy lifestyles within the community, and awareness and advocacy programs. Obesity and overweight screening can be recommended in children 6 years and above.

## 12. Conclusions

The escalating prevalence of obesity is a global concern demanding urgent attention. Its multifaceted impact on health, economy, and quality of life necessitates a comprehensive and collaborative response. Obesity's link to non-communicable diseases, psychological distress, and reduced productivity underscores the need for early screening, effective interventions, and public health campaigns. Viewing obesity as a chronic disease rather than a personal failing is pivotal to driving policy changes, medical advancements, and societal support. The Indian context further emphasizes the need for culturally tailored solutions. Stricter diagnostic criteria, considering ethnicity-specific factors, are imperative to identify individuals at risk accurately. Additionally, cost-effective interventions, such as lifestyle modifications and timely treatments, can mitigate the economic burden by improving health outcomes. Through collective efforts, we can effectively address the obesity epidemic and pave a path towards a healthier future.

## Figures and Tables

**Algorithm 1:**
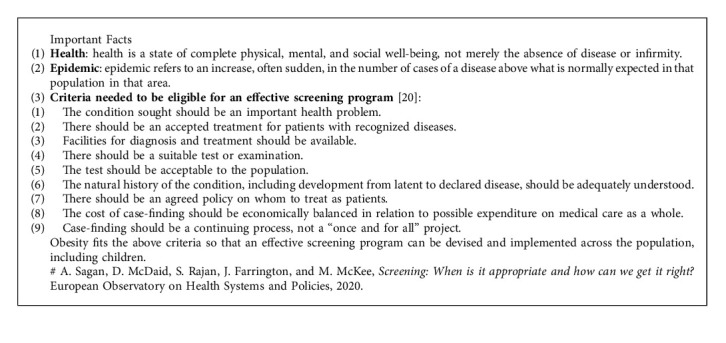
Important facts on health and obesity.

**Algorithm 2:**
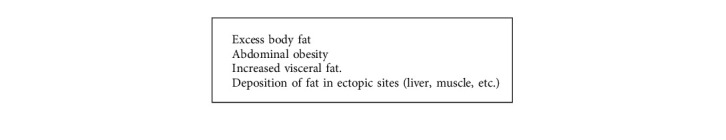
Unique features of obesity in Asian Indians.

**Table 1:** Definition of obesity as per various societies and organizations across the world.

Society	Definition
The Scottish Intercollegiate Guideline [9]	Obesity is defined as a disease process characterized by excessive body fat accumulation with multiple organ-specific consequences
The World Obesity Federation [4]	Obesity is a chronic relapsing disease process, defined by excessive adiposity that may impair health
American Medical Association [10]	Obesity is a disease state with multiple pathophysiological aspects requiring a range of interventions to advance obesity treatment and prevention
American Association of Clinical Endocrinologists and the American College of Endocrinology [11]	Obesity is a complex, adiposity-based chronic disease (ABCD), where management targets both weight-related complications and adiposity to improve overall health and quality of life
Obesity Canada [12]	Obesity is a chronic and progressive disease similar to diabetes or high blood pressure
American Society for Metabolic and Bariatric Surgery [13]	Obesity is a chronic progressive disease resulting from multiple environmental and genetic factors
American Heart Association [14]	Obesity is a multifactorial disease with a complex pathogenesis related to biological, psychosocial, socioeconomic, and environmental factors and heterogeneity in the pathways and mechanisms by which it leads to adverse health outcomes
European Association for the Study of Obesity [15]	Obesity is an adiposity-based chronic disease (ABCD). ABCD incorporates the characteristics of adiposity, including the total amount, distribution, and function of adipose tissue.
Royal College of Physicians [16]	Obesity is not a lifestyle choice caused by individual greed but a disease caused by health inequalities, genetic influences, and social factors
The Singapore Ministry of Health [17]	Obesity is a condition of excessive body fat with adverse effects on health and is an increasing global problem that contributes to chronic disease burden and healthcare costs
Malaysian Association for the Study of Obesity and Malaysian Diabetes Association [18]	Obesity is a complex, multifactorial condition characterized by excess body fat. It must be viewed as a chronic disorder that essentially requires perpetual care, support, and follow-up.
Endocrine Society of India [19]	Obesity is a chronic, relapsing progressive disease defined by excessive adiposity that may impair health. The management of obesity should follow the approach that is followed for any chronic disease, i.e., initiate interventions and keep titrating these with time in order to achieve our treatment goals.

**Table 2:** Other indicators to assess obesity.

Markers	Level in South Asians	Western population
*Waist circumference*		
Men	≥90 cm	≥102 cm
Women	≥80 cm	≥88 cm

*Waist-hip ratio*		
Men	≥0.9	≥0.9
Women	≥0.8	≥0.8

*Body fat percentage*		
Men	≥20%	≥25%
Women	≥33%	≥35%

**Table 3:** Obesity classification in different regions of the world.

	Age	Indicator	Normal weight	Overweight	Obese	Reference
*Adults*						
International	≥20 years	BMI (kg/m^2^)	18.5–25.99	≥25.00	≥30.00	Weir C. B., et al. BMI classification percentile and cut-off points. Treasure Island (FL): StatPearls publishing; 2023 Jan
				Pre-obese: 25–29.99	Class 1: 30.00–34.99	
					Class 2: 35.00–39.99	
					Class 3: ≥40.00	
South Asia	≥20 years	BMI (kg/m^2^)	>23.4	>25.2	≥26.6	Weir C. B., et al. BMI classification percentile and cut-off points. Treasure Island (FL): StatPearls publishing; 2023 Jan
Indian Consensus Group 2009	≥20 years	BMI (kg/m^2^)		23–24.9	≥25	Misra A, et al. J Assoc physicians India. 2009; 57 : 163–170
ESI 2022	≥18 years	BMI (kg/m^2^)	18.5–22.9	23–24.9	Obesity grade 1: 25–29.9	Madhu S. V., et al. Indian J endocrinol metab. 2022; 26 : 295–318
					Obesity grade 2: 30–34.9	
					Obesity grade 3: >35	
Clinical Practice Guidelines, Singapore	≥18 years	BMI (kg/m^2^)	18.5–24.9	25.0–29.9 (pre-obese)	Obese class I: 30–34.9	Clinical practice guidelines-obesity. Health promotion board, Singapore. 2016
					Obese class II: 35–39.9	
					Obese class III: ≥40	
Ministry of Health Malaysia, Malaysian Endocrine and Metabolic Society, Malaysian Association for the Study of Obesity, and Malaysian Diabetes Association	≥18 years	BMI (kg/m^2^)	18.5–22.9	≥23	Pre-obese: 23–27.4	Clinical practice guidelines management of obesity, 2^nd^ edition, 2023. Malaysia health technology assessment section
					Obese I: 27.5–34.9	
					Obese II: 35–39.9	
					Obese III: ≥40	

*Children*						
WHO 2006	0–60 months	BMI Z or WH Z	>-2 to ≤2 SD	>2 to ≤3 SD	>3 SD	Guideline: assessing and managing children at primary health-care facilities to prevent overweight and obesity in the context of the double burden of malnutrition: updates for the integrated management of childhood illness (IMCI). Geneva: World Health Organization; 2017
			At risk of overweight: >1 to ≤2 SD			
WHO 2007	5–19 years	BMI Z	>−2 to ≤1 SD	>1 to ≤2 SD	>2 SD	WHO. BMI-for-age (5–19 years). Accessed on 1^st^ September 2023 from https://www.who.int/tools/growth-reference-data-for-5to19-years/indicators/bmi-for-age
International Obesity Task Force	2–18 years	Growth curve for BMI at age 18		BMI = 25	BMI = 30	Cole T. J., et al. BMJ. 2000; 320 : 1240-1243
USA	2–19 years	BMI percentile	≥5^th^ to <85^th^	≥85^th^ to <95^th^	≥95^th^	Hampl S. E., et al. Paediatrics. 2023; 151: e2022060640

BMI, body mass index; EOSS, Edmonton Obesity Staging System; ESI, Endocrine Society of India; WHO, World Health Organization; WHZ, weight-for-height *z*-score; SD, standard deviation.

**Table 4:** The ABCDE barophenotypic characterization.

Barophenotype-ABCDE	
Adipose topography	Severity of obesity
	Style/pattern of weight distribution
	The swiftness of weight distribution
	Syndromic features

Barometric behavior	Diet
	Exercise/physical activity
	Addictions
	Stress and sleep

Comorbid status	Metabolic
	Medical
	Mechanical
	Mood

Dysfunctionality	Emotional
	Social
	Biomedical
	Biophysical

Enthusiasm and expectations	Weight loss expectation
	Willingness to change behavior
	Financial implications
	Social support

## Data Availability

No data were used to support this study.
