# Correction to “Defining and Diagnosing Obesity in India: A Call for Advocacy and Action”

**DOI:** 10.1155/jobe/9806198

**Published:** 2025-10-09

S. Kalra, N. Kapoor, M. Verma, et al., “Defining and Diagnosing Obesity in India: A Call for Advocacy and Action,” *Journal of Obesity* 2023 (2023): 4178121, https://doi.org/10.1155/2023/4178121.

In the article, the authors omitted the following funding statement:

“The manuscript has been supported by Novo Nordisk under independent publication support to Dr Sanjay Kalra.”

The conflicts of interest statement is therefore revised as follows:

“The authors declare that the publication of this article was supported by an independent grant from Novo Nordisk to facilitate open-access dissemination. No payments or financial incentives were provided to the authors. Novo Nordisk was not involved in the study design, data collection, analysis, manuscript preparation, or decision to publish.”

We apologize for this error.

